# New Insights Into the Complex Mutational Landscape of Sézary Syndrome

**DOI:** 10.3389/fonc.2020.00514

**Published:** 2020-04-21

**Authors:** Abu-Sayeef Mirza, Pedro Horna, Jamie K. Teer, Jinming Song, Ratilal Akabari, Mohammad Hussaini, Lubomir Sokol

**Affiliations:** ^1^Department of Internal Medicine, University of South Florida, Tampa, FL, United States; ^2^Division of Hematopathology, Mayo Clinic, Rochester, MN, United States; ^3^Department of Biostatistics and Bioinformatics, H. Lee Moffitt Cancer Center and Research Institute, Tampa, FL, United States; ^4^Department of Hematopathology and Laboratory Medicine, H. Lee Moffitt Cancer Center and Research Institute, Tampa, FL, United States; ^5^Department of Pathology, H. Lee Moffitt Cancer Center and Research Institute, Tampa, FL, United States; ^6^Department of Malignant Hematology, H. Lee Moffitt Cancer Center and Research Institute, Tampa, FL, United States

**Keywords:** cutaneous T-cell lymphoma, Sézary syndrome, whole exome sequencing, genomics, translational oncology

## Abstract

Sézary syndrome (SS) is a genetically and clinically distinct entity among cutaneous T-cell lymphomas (CTCL). SS is characterized by more aggressive disease compared to the most common indolent type of CTCL, mycosis fungoides. However, there are limited available genomic data regarding SS. To characterize and expand current mappings of the genomic landscape of CTCL, whole exome sequencing (WES) was performed on peripheral blood samples from seven patients with SS. We detected 21,784 variants, of which 21,140 were novel and 644 were previously described. Filtering revealed 551 nonsynonymous variants among 525 mutated genes−25 recurrent mutations and 1 recurrent variant. Several recurrently mutated genes crucial to pathogenesis pathways, including Janus kinase (JAK)/signal transducers and activators of transcription (STAT), peroxisome proliferator-activated receptors (PPAR), PI3K-serine/threonine protein kinases (AKT), and fibroblast growth factor receptors (FGFR), were identified. Furthermore, genetic mutations spanned both known and novel genes, supporting the idea of a long-tail distribution of mutations in lymphoma. Acknowledging these genetic variants and their affected pathways may inspire future targeted therapies. WES of a limited number of SS patients revealed both novel findings and corroborated complexities of the “long-tail” distribution of previously reported mutations.

## Introduction

Cutaneous T-cell lymphoma (CTCL) consists of a rare heterogeneous group of clonal T-cell lymphoproliferative disorders, including mycosis fungoides (MF) and Sézary syndrome (SS). Whereas MF is the most common indolent type of CTCL, SS exhibits more aggressive disease, which characteristically manifests with generalized erythroderma and circulating malignant cells in peripheral blood (1). Several studies have shown that there are distinct molecular pathogeneses and gene mutations between MF and SS (2, 3). In MF, gains in anti-apoptotic proteins and loss of cell cycle inhibitors result in increased cell survival. In SS, chromosomal alterations resulting in the dysregulation of the *MYC* oncogene and IL-2 receptor signaling pathway, the activation of cytokine pathways, and the inhibition of *P53* accounts for the increased cell proliferation and leukemic behavior observed in patients with this disease (3, 4).

There are limited studies on whole exome sequencing (WES) of CTCL, yielding varying results. In one study, WES analyses of 42 CTCL cases, including 25 SS and 8 MF cases, showed highly prevalent chromosomal deletions involving the *TP53, RB1, PTEN, DNMT3A*, and *CDKN1B* tumor suppressors, which broadly implicates epigenetic regulation and signaling (5). In another study, whole genome and transcriptome next-generation sequencing analyses of nine patient samples showed copy variations in 8q (*MYC, TOX*), 17p (*TP53, NCOR1*), 10q (*PTEN, FAS*), 2p (*DNMT3A*), 11q (*USP28*), and 9p (*CAAP1*), but no recurrent rearrangements were identified (6). The largest retrospective WES analysis of CTCL to date included 220 patients with CTCL (including 186 SS patients and 25 MF patients) and used publicly available sequencing data across nine studies (7). This study identified 55 putative driver genes and implicated 17 novel gene mutations involving pathways that affect chromatin remodeling (*BCOR, KDM6A, SMARCB1, TRRAP*), immune surveillance (*CD58, RFXAP*), MAPK signaling (*MAP2K1, NF1*), NF-κB signaling (*PRKCB, CSNK1A1*), PI-3-kinase signaling (*PIK3R1, VAV1*), RHOA/cytoskeleton remodeling (*ARHGEF3*), RNA splicing (*U2AF1*), T-cell receptor signaling (*PTPRN2, RLTPR*), and T-cell differentiation (*RARA*) (7). Point mutations, single gene alterations, and copy number alterations in SS represent genomic diversity involving multiple pathways, such as T-cell receptor signaling, NF-kB and JAK/STAT pathways, apoptosis control, chromatin remodeling, and DNA damage response (8). Therefore, the clinical heterogeneity of MF and SS cannot be solely explained by known mutations.

Other WES analyses, such as those performed by Choi et al. (9) further confirmed that CTCL genomic diversity involves multiple pathways, including T-cell receptor signaling, NF-kB and JAK/STAT pathways, apoptosis control, chromatin remodeling, and DNA damage response.

Given these limited studies and varying results, we used WES to further expand and characterize the genomic landscape of SS. The main aim of the present study was to validate current understandings of SS genomics and identify previously unreported novel mutations.

## Methods

Eight patients with SS were identified through institutional query following scientific review committee and institutional review board approval (MCC17922). Patient samples were collected from peripheral blood and cryopreserved. Neoplastic cells from all samples were CD3^+^ and accounted for >50% of mononuclear cells, as assessed by flow cytometry. CD3^−^ mononuclear cells sorted by flow cytometry were used as germline controls. DNA was extracted in accordance with standard protocols of the diagnostic molecular laboratory at H. Lee Moffitt Cancer Center and Research Institution (Tampa, FL, USA). WES analyses were performed at Hudson Alpha Institute for Biotechnology (Huntsville, AL, USA) on samples from eight patients. One patient lacked a paired normal sample and was excluded from the analysis.

Library prep was performed by using NimbleGen SeqCap EZ Exome Library v3.0. Sequencing was performed on HiSeq X sequencers (Illumina, San Diego, CA) at 100× for tumor sample and 30× for paired normal sample. A combined pipeline using Strelka (Illumina, San Diego, CA) and Mutect (Broad Institute, Cambridge, MA) was used to perform bioinformatics analyses. Variants with a minor allele frequency >1% in germline databases (1,000 Genomes Project) were excluded. Intronic, untranslated regions, and synonymous variants were also excluded. WEB-based GEne SeT AnaLysis Toolkit (WebGestalt) was used to perform enrichment analyses, and Kyoto Encyclopedia of Genes and Genomes (KEGG), Reactome, and Wikipathways were used to perform pathway analyses.

## Results

WES was finalized on paired tumor/normal samples from seven unique patients (clinical characteristics are described in Table 1). After filtering, mutations that were present in both total mononuclear cells and CD3^−^ mononuclear cells were assumed to be germline and were excluded. WES analyses detected 21,784 somatic mutations across seven samples. Twenty-six percent of variants detected were found to have no protein changes (synonymous mutations).

**Table 1:** Clinical characteristics.

**Patient**	**Stage**	**%TBSA**	**Sezary load/μL**	**No. of systemic therapies**	**Disease status**	**Deceased**	OS^*^ (months)
1	T4N1M0B2	50	10,223	5	CR	N	80
2	T4NxM0B2	100	1,867	6	PD	Y	43
3	T4NxM0B2	50	53,000	5	PD	Y	15
4	T4N1M0B2	100	19,600	1	SD	Y	12
5	T4N2M0B2	100	7,710	6	PR	N	82
6	T3N1M0B2	50	2,737	5	SD	Y	33
7	T2N1M0B2	80	12,070	3	PD	N	35

*Seven patients with Sezary Syndrome and their clinical characteristics*.

*TNMB staging, tumor-node-metastasis-blood; %TBSA, percentage of total body surface area*.

* *Overall survival reflects the last day of follow-up for patients who are not deceased*.

Further filters were applied to exclude synonymous variants, variants that were present at >1% in 1,000 genomes, and variants with a quality score <3. WES revealed 551 non-synonymous variants distributed across 525 genes, including 478 missense mutations and 73 nonsense, splicing, or frameshift mutations. The following recurrent variants were detected: *C7orf42* p.T187P (two patient samples), *PTPN4* splicing variant c.1814-2A (one patient sample), and *ANKRD46* splicing variant c.312-2A>T (one patient sample). The *PTPN4* and *ANKRD46* variants were considered to be sequencing artifacts because of their presence in a homopolymer region and presence in control specimens. The *C7orf42* p.T187P variant was also suspected to be a technical artifact but was retained for having met predetermined quality criteria.

Out of the 21,784 somatic variants detected, 21,140 (97%) were novel variants and 644 were previously described variants based on Ensemble analyses; 86.8% of mutations were missense and 13.3% of mutations were truncating. The 525 genes affected by nonsynonymous somatic changes (single nucleotide variants and indels), along with genes affected by copy number loss or gain (i.e., *TP53, STK11, MYC, MAP2K4, GNA11*), were further analyzed (Supplementary Table 1). Recurrently mutated genes (mutated in >2 patient samples) included *ANK3, CAMSAP1, C7orf42, CSMD1, DH11, FAT1, FLAD1, FLNB, FRAS1, GLUD2, GRIA2, ITGB8, KCND2, LRP1B, LRP2, MYH4, NRCAM, OR2L2, PAPPA2, PCLO, PKHD1L1, UNC13C, VWA3B*, and *XIRP2*. *LRP2* was mutated in three patient samples. Certain genes were not mutated across patient samples but harbored multiple mutations in the same patient sample.

Among the genes that were mutated more than once (regardless of whether the mutation occurred twice in the same patient), the most frequently mutated genes were *GLUD2, LRP2*, and *PABPC3* (Table 2). Copy number variant analyses showed three samples with *TP53* loss, three samples with *STK11* loss, three samples with *MYC* gain, three samples with *MAP2K4* loss, and three samples with *GNA11* loss (Supplementary Figure 1).

**Table 2:** Recurrent mutations.

**Gene**	**No. of mutations in gene**	**Sézary 1**	**Sézary 2**	**Sézary 3**	**Sézary 4**	**Sézary 5**	**Sézary 6**	**Sézary 7**
ANK3	2							
C7orf42	1							
CAMSAP1	2							
CSMD1	2							
DH11	1							
FAT1	2							
FLAD1	1							
FLNB	2							
FRAS1	2							
**GLUD2**^a^	**6**							
GRIA2	2							
ITGB8	2							
KCND2	2							
LRP1B	1							
**LRP2**	**3**							
MYH4	2							
NRCAM	2							
OR2L2	2							
PAPPA2	2							
PCLO	2							
PKHD1L1	2							
TACR3	2							
UNC13C	2							
VWA3B	2							
XIRP2	2							

*All genes found to be mutated in two or more patients and/or genes with multiple mutations within the same gene (bolded genes discussed further in text). Dark blue indicates a missense mutation; light blue indicates a truncating mutation. Genes mutated more than once had multiple mutations within the same gene, whereas recurrently mutated genes were mutated twice or more in >1 SS patient*.

a *Bolded genes discussed in the text in more detail*.

*mut, mutated; trunc, truncated*.

WEB-based GEne SeT AnaLysis Toolkit (WebGestalt) gene ontology functional database was used to investigate the role of the identified altered genes. GoSlim summary divided genes into biological processes, cellular components, and molecular function categories. Four hundred eighty-seven genes and 42 IDs were excluded from the analysis, as they were inadequately mapped to Entrez Gene IDs. A minimum of five genes per category were required for binning. Genes in the biological processes category were most commonly involved in biological regulation, metabolic processes, and response to stimuli. The molecular functions of the genes involved were most commonly protein binding, iron binding, and nucleic acid binding.

WebGestalt enrichment analyses showed enrichment of genes in the glutamate receptor signaling pathway (enrichment ratio = 4.81, *P* = 9.83 × 10^−5^). Similar overrepresentation analyses (ORA) performed using KEGG demonstrated enrichment of genes in the PI3K-AKT signaling pathway (*R* = 1.98; *P* = 5.43 × 10^−3^) (Supplementary Figure 2). This finding was confirmed by Wikipathways analyses. Overall mapping of mutated genes to cancer pathways showed that pathways, including the PPAR and JAK/STAT pathways, were mainly involved in providing proliferation signals. KEGG gene mapping further confirmed the involvement of the PPAR and JAK/STAT pathways. Reactome pathway analyses reconfirmed that the PI3K pathway and signal transduction particularly involved the fibroblast growth factor receptor (FGFR; Supplementary Figure 3).

## Discussion

WES analyses were used to elucidate the molecular biology of SS and its genomic landscape. Despite having a limited sample size, this study validated the genomic diversity of SS, characterized by the disease's long-tail distribution of genomic mutations. By focusing on recurrent gene mutations in multiple samples from seven SS patients, we highlighted both novel and known mutations and pathways.

Multiply mutated genes included *LRP2, GLUD2*, and *PABC3*. *LRP2* is a member of the *LDLR* family and an endocytic receptor. LRP2 is expressed on the apical surface of absorptive epithelial cells and facilitates internalization of different ligands, such as lipoproteins, sterols, vitamin-binding proteins, hormones, signaling molecules, and extracellular matrix proteins (10). Once internalized, these ligands undergo lysosomal degradation or transcytosis (10). *LRP2* can also form complexes with cubilin, which can be inhibited by sodium maleate (11, 12). *LRP2* expression has been shown to be crucial for cell maintenance in malignant melanoma, and siRNA-mediated reduction of *LRP2* in melanoma cells significantly decreased melanoma cell proliferation and survival rates (12). *LRP2* gene polymorphisms have also been studied in regards to prostate cancer given the influence of steroid hormone uptake by endocytic receptors in prostate epithelial cells (13).

*GLUD2* mutations have not been previously mentioned in regards to SS. *GLUD2* is a housekeeping gene that is widely expressed and plays a crucial role in glutamate metabolism (14). RNA sequencing of triple-negative breast cancer samples has shown *GLUD2* variant mutations (15).

Another multiply mutated gene was *PABPC3*, which is known to be an important RNA-binding protein in the translational regulation of mRNAs in spermatogenesis (16). WES analyses of six follicular thyroid cancer cell lines revealed *PABPC3* to be a recurrently mutated cancer driver gene (17). These findings support the idea of a potential pathogenic role of these mutations in SS.

Our study confirmed the dysregulation of the PI3K/AKT pathway in SS, as previously reported (18). The PI3K/AKT pathway is implicated in multiple malignancies and is involved with tumor suppression when antagonized by *PTEN*. PI3K overexpression is an oncogenic factor in squamous cell carcinomas and is considered to be a therapeutic target (19). Interestingly, the cytokine IL-31R was found to be involved with the PI3K/AKT pathway in relation to the pathogenesis of intense pruritis among MF/SS patients (20, 21). Furthermore, AKT activation as a proxy for hyperproliferation and growth was more often found in SS skin cells than in circulating SS cells, suggesting a molecular pathogenesis of cutaneous manifestations (22).

Activated AKT is considered to be a survival factor for inhibiting apoptosis via phosphorylation of several key targets, including FOXO transcription factors (23). Inactivation of various elements of the FOXO family has been reported in the development of multiple myeloid leukemias and oncogenesis in pre-B acute lymphoblastic leukemia (24). In SS patients, *FOXO1A* was found to be downregulated, resulting in loss of control mechanisms for cell cycle, cell death, cell metabolism, and oxidative stress (25).

Cristofoletti et al. discovered *PTEN* to be deleted in 36% of patients with SS and downregulated in almost all SS samples (*n* = 44) (22). The *PTEN* gene (locus at 10q23) may enhance resistance to apoptosis in SS cells by downregulating *FOXO3a*, thereby contributing to malignant expansion (22). In mice studies, T-cell *PTEN* deletions have resulted in the development of CD4^+^ T-cell lymphomas (26). In MF and SS, PI3K inhibition has been shown to potentiate HDAC-inhibitor antitumor activity (27). However, the role of PIK3 inhibition as a single agent or adjuvant therapy remains to be elucidated.

Reactome analyses showed FGFR signaling involvement. Mutations in the FGFR family of proteins have been reported in regards to several malignancies (28). In certain malignancies, FGFR signaling inhibition can result in antiproliferative and/or proapoptotic effects, and FGFRs can activate several oncogenic pathways, such as STAT-dependent signaling, Ras-dependent MAPK, and Ras-independent PI3K/AKT signaling pathways (28). Several targeted therapies against FGFR, ranging from monoclonal antibodies to specific inhibitors, are currently being studied in phase 1/2 clinical trials for the treatment of several types of cancer (28, 29). As anti-FGFR therapy for CTCL has not been studied, our data provide the basis for further therapeutic investigation of this therapy.

We also detected a 17p deletion in three out of seven SS patients. This finding was consistent with that of Prasad et al. who reported a *TP53* gene deletion and/or mutation in 58% of SS patients (30). Given the positive regulatory relationship between *TP53* and *PTEN*, the combined dysfunction of *PTEN* and *TP53* is suspected to contribute to the genetic instability of SS cells. This genetic instability facilitates chromosomal alterations, namely losses, gains, and rearrangements (22). The recurrent nature of *TP53* aberrations in SS patients may constitute a distinct clinical subtype (8). Pharmacological activation of *P53* may be considered in the future if it is combined with traditional chemotherapies (31).

Several studies have shown large regions of chromosomes affected by recurrent copy number variations in regions of known oncogenes (4, 32). Our copy number variant analyses showed that several genes had losses in three tumor samples (*TP53, STK11, MAP2K4, GNA11*), and one gene had a gain in three tumor samples (*MYC*).

*STK11* loss was found in three out of seven patients in our study. *STK11* is a tumor suppressor gene that plays a crucial role in cell growth regulation and apoptosis (33). *STK11* kinase activity elimination is associated with the Peutz-Jeghers syndrome and an elevated cancer risk (34). In a genotypically and phenotypically distinct subset of lung adenocarcinoma cell lines, *STK11* inactivation was found to be common and to attenuate the PI3K/AKT pathway (35). In one case report, a patient with triple-negative breast cancer with a point mutation in *STK11* with loss of heterozygosity had a near-complete response with everolimus therapy. This response may be explained by the relationship between *STK11* and the PI3K/AKT/mTOR signaling pathway (36).

Copy number variation analyses, as reported by Lee et al. (37) also showed loss of *STK11, MAP2K4, GNA11*, and *MYC* gene gain-of-function aberrations. The observed loss in this study supports the theory that the more aggressive behavior of SS involving blood, skin, and lymph nodes may be facilitated via *MYC* dysregulation (4).

*MAP2K4* loss was seen in three out of seven of our patients. The MAPK pathway, similar to the PI3K pathway, contributes to oncogenesis by cell proliferation and antiapoptotic activity (38). The loss-of-function mutation in *MAP2K4* has been observed to be highly frequent in several cancers (37–40). Xue et al. (38) demonstrated that inactivating mutations in *MAP2K4* increased cell line sensitivity to MEK inhibitor therapy, thereby enhancing response to the therapy.

*GNA11 loss* was observed in three out of seven patients. *GNA11* is considered to be an early driver mutation in leptomeningeal and uveal melanomas (41, 42). Over 80% of uveal melanomas are known to have mutations in *GNA11* (43). Using *GNA11* mutated melanoma cell lines, MEK inhibitors to suppress MAPK pathways, and suppressing protein kinase C led to the synergistic inhibition of proliferation (44).

*MYC* gene gain-of-function aberrations from translocation, amplification, or overexpression are common in tumorigenesis (45) and are associated with *TP53* loss mutation. Both MYC gain and TP53 loss are inversely related to poor 5-year overall survival rates (46). When the proto-oncogene *MYC* is overactivated, it triggers an antioncogenic mitosis-differentiation checkpoint in human epidermal keratinocytes, resulting in impaired cell division, and squamous differentiation (47). Our data appear to support the hypothesis that disseminated leukemic behavior of SS may be affected by *MYC* dysregulation (4).

Our study underscores the need to sequence more SS cases, given the genomic heterogeneity of this disease and the potential for identifying targetable therapies. To further understand the implications of the genomic alterations in SS described in this study, functional characterization of the detected genetic alterations, prospective studies using larger sample sets, therapeutic clinical trials with targeted agents, and correlation with outcomes are all needed.

## Data Availability Statement

All datasets generated for this study are included in the article/Supplementary Material.

## Ethics Statement

The studies involving human participants were reviewed and approved by Scientific Review Committee of Moffitt Cancer Center, Institutional Review Board of Moffitt Cancer Center (MCC17922). The patients/participants provided their written informed consent to participate in this study.

## Author Contributions

A-SM: developed manuscript, developed figures, and data analysis. PH: data analysis. JT: bioinformatics, data analysis. JS: hematopathology research, reviewed paper. RA: hematopathology research, sample preparation, and quality control. MH: hematopathology research, data analysis, IRB proposal/renewal, and developed supplemental figures. LS: malignant hematology research, clinician, principal investigator, and reviewed paper.

## Conflict of Interest

LS served as a coinvestigator on MAVORIC and ALCANZA studies and received research funding from Spectrum Pharmaceuticals, Celgene, and Seattle Genetics. The remaining authors declare that the research was conducted in the absence of any commercial or financial relationships that could be construed as a potential conflict of interest.
