# Supplementary material for: New Insights Into the Complex Mutational Landscape of Sézary Syndrome
Source: Front Oncol. 2020 Apr 21;10:514. doi: 10.3389/fonc.2020.00514 (PMC7186303; doi:10.3389/fonc.2020.00514)
Supplement: Supplementary file 1 [file Data_Sheet_1.DOCX]

**Supplementary Figure 1.** Copy number analysis demonstrating TP53 loss (chromosome 17) among three tumor samples.


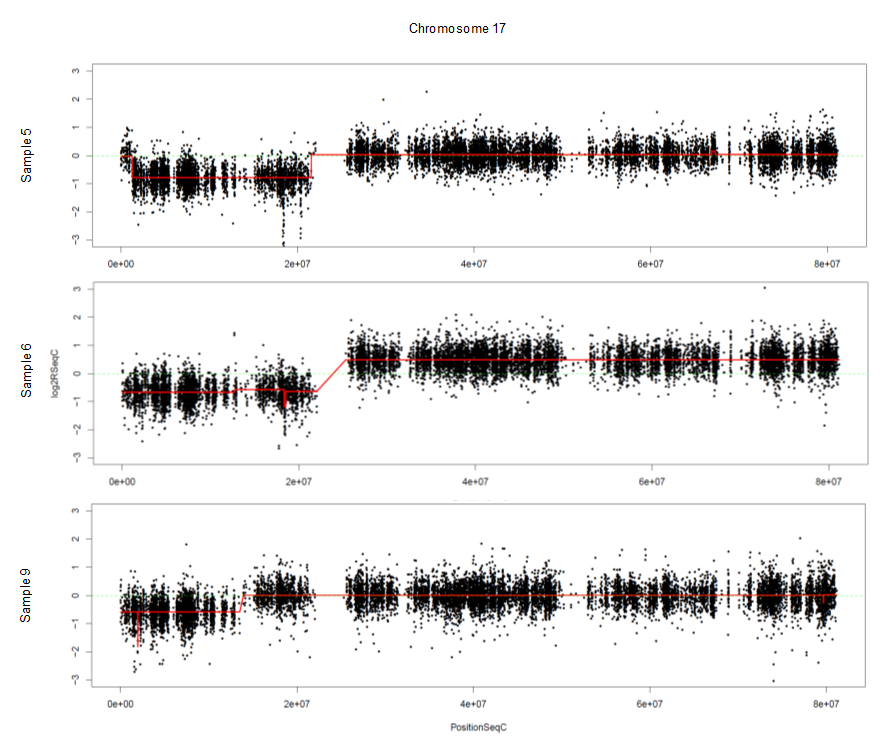


**Supplementary Figure 2.** ORA Enrichment analysis summary.

Supplementary Figure 3: **Reactome Pathway analysis**.

**Genome-wide overview of pathway analysis**. The center of each Reactome pathway is the root and each step from the center represent the next level lower in the pathway hierarchy. The color gradients represent the degree of overrepresentation of the pathway. Signal transduction, extracellular matrix organization, and cell cycle pathways are implicated amongst others.


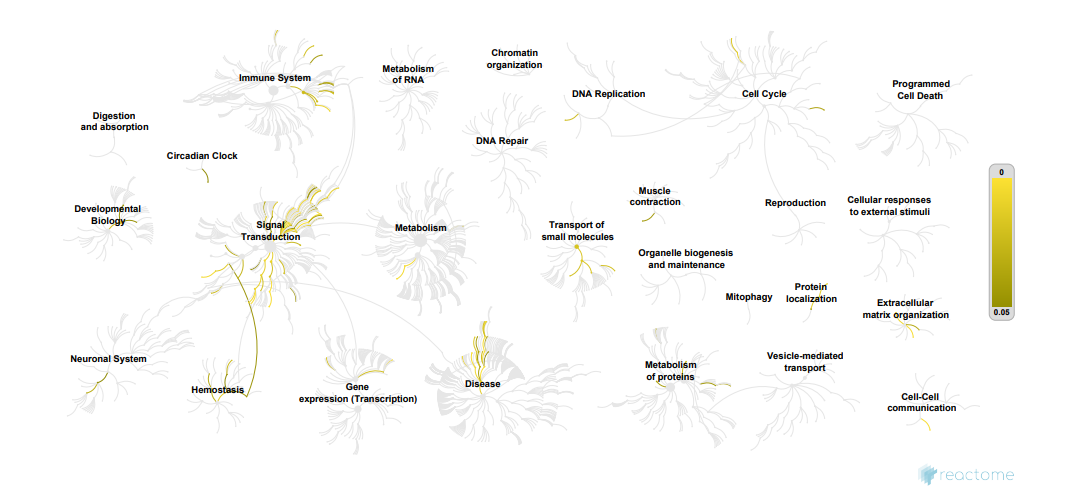


Supplementary Table 1. A list of 525 genes affected by nonsynonymous somatic changes (single nucleotide variants and indels), along with genes affected by copy number loss or gain.

| 1 | AATK |
| --- | --- |
| 2 | ABCA2 |
| 3 | ABCA4 |
| 4 | ABCA9 |
| 5 | ABCC1 |
| 6 | ABCC10 |
| 7 | ABCC12 |
| 8 | ABCC4 |
| 9 | ACSM2A |
| 10 | ACSM2B |
| 11 | ACTB |
| 12 | ACTRT2 |
| 13 | ADAM12 |
| 14 | ADAM19 |
| 15 | ADAMTSL4 |
| 16 | ADARB1 |
| 17 | ADARB2 |
| 18 | ADCY7 |
| 19 | ADH1A |
| 20 | AFAP1L1 |
| 21 | AIM1L |
| 22 | AKAP9 |
| 23 | AKR1A1 |
| 24 | ALDH2 |
| 25 | ALOX5 |
| 26 | AMFR |
| 27 | ANK3 |
| 28 | ANKDD1A |
| 29 | ANKRD10 |
| 30 | ANKRD30A |
| 31 | ANKRD46 |
| 32 | ANKS6 |
| 33 | ANO8 |
| 34 | APOB |
| 35 | APOBEC3F |
| 36 | ARHGAP29 |
| 37 | ARHGAP6 |
| 38 | ART5 |
| 39 | ASCC3 |
| 40 | ASNS |
| 41 | ATP2A3 |
| 42 | ATP2B2 |
| 43 | BAZ1B |
| 44 | BBS9 |
| 45 | BBX |
| 46 | BCAM |
| 47 | BFSP1 |
| 48 | BNC2 |
| 49 | BRK1 |
| 50 | BRWD1 |
| 51 | C10orf11 |
| 52 | C14orf37 |
| 53 | C15orf59 |
| 54 | C16orf95 |
| 55 | C16orf96 |
| 56 | C17orf28 |
| 57 | C17orf63 |
| 58 | C19orf21 |
| 59 | C1QTNF2 |
| 60 | C1orf111 |
| 61 | C1orf124 |
| 62 | C2orf54 |
| 63 | C3orf70 |
| 64 | C7orf42 |
| 65 | C9orf3 |
| 66 | CAC1A |
| 67 | CAC1E |
| 68 | CAC1S |
| 69 | CADM1 |
| 70 | CALD1 |
| 71 | CAMSAP1 |
| 72 | CAMSAP3 |
| 73 | CAND2 |
| 74 | CAPN2 |
| 75 | CARD6 |
| 76 | CCDC103 |
| 77 | CCDC110 |
| 78 | CCDC149 |
| 79 | CCDC36 |
| 80 | CCDC38 |
| 81 | CCNB1 |
| 82 | CCNB3 |
| 83 | CD163L1 |
| 84 | CD28 |
| 85 | CDH12 |
| 86 | CDH18 |
| 87 | CDH20 |
| 88 | CDKAL1 |
| 89 | CDX2 |
| 90 | CEACAM19 |
| 91 | CECR6 |
| 92 | CELSR3 |
| 93 | CEP112 |
| 94 | CERS5 |
| 95 | CETN1 |
| 96 | CGNL1 |
| 97 | CHMP4C |
| 98 | CLASP2 |
| 99 | CLCA1 |
| 100 | CLEC3A |
| 101 | CLINT1 |
| 102 | CLVS2 |
| 103 | CNTN1 |
| 104 | CNTP1 |
| 105 | CNTP2 |
| 106 | CNTP3B |
| 107 | COL1A1 |
| 108 | COL20A1 |
| 109 | COL24A1 |
| 110 | COL5A2 |
| 111 | CORO2B |
| 112 | CPNE8 |
| 113 | CPS1 |
| 114 | CRISP1 |
| 115 | CRLS1 |
| 116 | CRYGA |
| 117 | CSMD1 |
| 118 | CSMD3 |
| 119 | CTTNBP2 |
| 120 | CWH43 |
| 121 | CYLC1 |
| 122 | CYP1A1 |
| 123 | DAPK1 |
| 124 | DCAF13 |
| 125 | DCHS2 |
| 126 | DCTPP1 |
| 127 | DDX25 |
| 128 | DDX60 |
| 129 | DHX40 |
| 130 | DHX58 |
| 131 | DLEC1 |
| 132 | DMD |
| 133 | DH1 |
| 134 | DH10 |
| 135 | DH11 |
| 136 | DH17 |
| 137 | DH3 |
| 138 | DH8 |
| 139 | DI1 |
| 140 | DNMT3A |
| 141 | DOCK4 |
| 142 | DOK3 |
| 143 | DOPEY2 |
| 144 | DPEP2 |
| 145 | DPYSL3 |
| 146 | DPYSL5 |
| 147 | DSG4 |
| 148 | DST |
| 149 | DUSP27 |
| 150 | DYRK3 |
| 151 | EAF2 |
| 152 | EF2 |
| 153 | EFTUD2 |
| 154 | EIF3B |
| 155 | EIF5B |
| 156 | ELFN1 |
| 157 | EM |
| 158 | EPHA6 |
| 159 | EPHA8 |
| 160 | ERN1 |
| 161 | ESF1 |
| 162 | ESRRG |
| 163 | ESYT2 |
| 164 | ETV4 |
| 165 | EVL |
| 166 | F11 |
| 167 | FAM116A |
| 168 | FAM155B |
| 169 | FAM19A5 |
| 170 | FAM48A |
| 171 | FAM55D |
| 172 | FAM63B |
| 173 | FAM75D1 |
| 174 | FAM90A1 |
| 175 | FARP1 |
| 176 | FAS |
| 177 | FASN |
| 178 | FAT1 |
| 179 | FAT3 |
| 180 | FAT4 |
| 181 | FBN1 |
| 182 | FBXO8 |
| 183 | FBXW4 |
| 184 | FCHO2 |
| 185 | FER1L6 |
| 186 | FGD5 |
| 187 | FGF14 |
| 188 | FGF16 |
| 189 | FGF23 |
| 190 | FGFR2 |
| 191 | FGGY |
| 192 | FHOD3 |
| 193 | FLAD1 |
| 194 | FLNB |
| 195 | FLYWCH1 |
| 196 | FMO3 |
| 197 | FOXN1 |
| 198 | FRAS1 |
| 199 | FREM3 |
| 200 | FRMD4A |
| 201 | FRYL |
| 202 | FSCB |
| 203 | FUBP1 |
| 204 | FZD3 |
| 205 | GABPB2 |
| 206 | GABRA5 |
| 207 | GABRE |
| 208 | GABRG1 |
| 209 | GAGE13 |
| 210 | GAPDHS |
| 211 | GAPVD1 |
| 212 | GDF3 |
| 213 | GDPD3 |
| 214 | GLT1D1 |
| 215 | GLUD2 |
| 216 | GNLY |
| 217 | GON4L |
| 218 | GPATCH8 |
| 219 | GPR161 |
| 220 | GPR20 |
| 221 | GPR98 |
| 222 | GPT2 |
| 223 | GRIA2 |
| 224 | GRIA3 |
| 225 | GRIA4 |
| 226 | GRIK5 |
| 227 | GRIN3B |
| 228 | GRM7 |
| 229 | GTF3C1 |
| 230 | H3F3C |
| 231 | HAO1 |
| 232 | HDAC4 |
| 233 | HEATR4 |
| 234 | HEATR7B2 |
| 235 | HECW1 |
| 236 | HGC6.3 |
| 237 | HIPK2 |
| 238 | HIRIP3 |
| 239 | HIST1H1E |
| 240 | HIST1H2AH |
| 241 | HLA-A |
| 242 | HMBOX1 |
| 243 | HOPX |
| 244 | HOXC9 |
| 245 | HTN3 |
| 246 | HTR4 |
| 247 | ID3 |
| 248 | IGF2BP2 |
| 249 | IGFN1 |
| 250 | IGSF21 |
| 251 | IL20RA |
| 252 | INPP5E |
| 253 | IP6K3 |
| 254 | ITGB2 |
| 255 | ITGB8 |
| 256 | IWS1 |
| 257 | JAKMIP1 |
| 258 | JPH2 |
| 259 | KALRN |
| 260 | KCND2 |
| 261 | KCNT2 |
| 262 | KDR |
| 263 | KIAA0182 |
| 264 | KIAA0513 |
| 265 | KIAA1211 |
| 266 | KIAA1377 |
| 267 | KIAA1462 |
| 268 | KIAA1751 |
| 269 | KIAA2018 |
| 270 | KIF3B |
| 271 | KLC3 |
| 272 | KLHL11 |
| 273 | KLHL32 |
| 274 | KRT13 |
| 275 | KRT26 |
| 276 | KRT83 |
| 277 | KRTAP4-3 |
| 278 | KSR1 |
| 279 | LAMA1 |
| 280 | LAMA2 |
| 281 | LAMA5 |
| 282 | LAMC1 |
| 283 | LCA5L |
| 284 | LCN10 |
| 285 | LDHD |
| 286 | LGSN |
| 287 | LHFPL3 |
| 288 | LILRA1 |
| 289 | LOC388946 |
| 290 | LRFN5 |
| 291 | LRIT2 |
| 292 | LRP1B |
| 293 | LRP2 |
| 294 | LRRC16A |
| 295 | LRRC52 |
| 296 | LRRC69 |
| 297 | LRRIQ4 |
| 298 | LTB |
| 299 | LTBP1 |
| 300 | LY75 |
| 301 | LY75-CD302 |
| 302 | LYSMD4 |
| 303 | MAGI1 |
| 304 | MAP3K4 |
| 305 | MAP3K9 |
| 306 | MAPKBP1 |
| 307 | MDGA2 |
| 308 | MECOM |
| 309 | MED12 |
| 310 | MEGF6 |
| 311 | MEP1A |
| 312 | METTL14 |
| 313 | MGAT4C |
| 314 | MKI67 |
| 315 | MLF1IP |
| 316 | MLPH |
| 317 | MMP13 |
| 318 | MNS1 |
| 319 | MORC3 |
| 320 | MSH4 |
| 321 | MSLN |
| 322 | MTCP1 |
| 323 | MTMR3 |
| 324 | MTTP |
| 325 | MUC17 |
| 326 | MUC5B |
| 327 | MUSK |
| 328 | MXRA5 |
| 329 | MYH4 |
| 330 | MYO5C |
| 331 | MYOCD |
| 332 | ALADL2 |
| 333 | T2 |
| 334 | V3 |
| 335 | NBEA |
| 336 | NBPF10 |
| 337 | NCAM1 |
| 338 | NCAPG2 |
| 339 | NCOA6 |
| 340 | NNMT |
| 341 | NRCAM |
| 342 | NTRK3 |
| 343 | OAT |
| 344 | OPRM1 |
| 345 | OR10R2 |
| 346 | OR2A25 |
| 347 | OR2L2 |
| 348 | OR2T4 |
| 349 | OR2V2 |
| 350 | OR4A15 |
| 351 | OR4K13 |
| 352 | OR5AR1 |
| 353 | OR6K6 |
| 354 | OR8H3 |
| 355 | OSMR |
| 356 | OSTBETA |
| 357 | OTOF |
| 358 | PABPC3 |
| 359 | PADI3 |
| 360 | PAPPA2 |
| 361 | PARD6G |
| 362 | PAX8 |
| 363 | PCDH17 |
| 364 | PCDH7 |
| 365 | PCDHB1 |
| 366 | PCDHB10 |
| 367 | PCDHGA9 |
| 368 | PCLO |
| 369 | PCSK5 |
| 370 | PDZD7 |
| 371 | PHC1 |
| 372 | PHF14 |
| 373 | PHF3 |
| 374 | PIEZO2 |
| 375 | PIGY |
| 376 | PITPNC1 |
| 377 | PKD1L1 |
| 378 | PKHD1L1 |
| 379 | PLAGL2 |
| 380 | PLCXD3 |
| 381 | PLCZ1 |
| 382 | PLD1 |
| 383 | PLEC |
| 384 | PLX1 |
| 385 | PNLIP |
| 386 | PNLIPRP3 |
| 387 | POF1B |
| 388 | POLE2 |
| 389 | PPAP2C |
| 390 | PRDM16 |
| 391 | PRDM9 |
| 392 | PRKAG3 |
| 393 | PRKD1 |
| 394 | PRRC2A |
| 395 | PRSS12 |
| 396 | PRSS36 |
| 397 | PTN |
| 398 | PTPN4 |
| 399 | PTPN6 |
| 400 | QSOX2 |
| 401 | RAB22A |
| 402 | RAB43 |
| 403 | RADIL |
| 404 | RALGAPA2 |
| 405 | RAPSN |
| 406 | RBFOX1 |
| 407 | RBM11 |
| 408 | RBM12 |
| 409 | RBM44 |
| 410 | RBM48 |
| 411 | RBMXL1 |
| 412 | RDH13 |
| 413 | REG3G |
| 414 | REXO1 |
| 415 | RFC3 |
| 416 | RIN2 |
| 417 | RIPK4 |
| 418 | RNF187 |
| 419 | RNF217 |
| 420 | RTTN |
| 421 | RXRG |
| 422 | SALL1 |
| 423 | SCNN1G |
| 424 | SCYL2 |
| 425 | SDCBP |
| 426 | SEL1L |
| 427 | SEMA6D |
| 428 | SFMBT2 |
| 429 | SH2D5 |
| 430 | SHANK1 |
| 431 | SIM1 |
| 432 | SIM2 |
| 433 | SIPA1L2 |
| 434 | SLC13A3 |
| 435 | SLC15A5 |
| 436 | SLC1A1 |
| 437 | SLC25A2 |
| 438 | SLC28A2 |
| 439 | SLC2A4 |
| 440 | SLC41A1 |
| 441 | SMOC2 |
| 442 | SMPDL3A |
| 443 | SOX17 |
| 444 | SPEG |
| 445 | SPHKAP |
| 446 | SPP1 |
| 447 | SPSB4 |
| 448 | SPTA1 |
| 449 | SRPX2 |
| 450 | SRRM4 |
| 451 | SSPO |
| 452 | STAB1 |
| 453 | STARD3 |
| 454 | STAT5B |
| 455 | SVEP1 |
| 456 | SYS1 |
| 457 | SYT3 |
| 458 | SYT6 |
| 459 | TACC2 |
| 460 | TACR3 |
| 461 | TAGLN2 |
| 462 | TAL1 |
| 463 | TARDBP |
| 464 | TARS |
| 465 | TBC1D8B |
| 466 | TCP11 |
| 467 | TECTA |
| 468 | TENC1 |
| 469 | TET1 |
| 470 | TFAP2A |
| 471 | TFDP3 |
| 472 | TGFBI |
| 473 | TLL1 |
| 474 | TLR4 |
| 475 | TMEM44 |
| 476 | TMSB4X |
| 477 | TNFRSF21 |
| 478 | TOX |
| 479 | TP53 |
| 480 | TPST1 |
| 481 | TRIM47 |
| 482 | TSPAN8 |
| 483 | TSPEAR |
| 484 | TTC38 |
| 485 | TTC5 |
| 486 | TTLL4 |
| 487 | TTN |
| 488 | TUBGCP6 |
| 489 | ULK1 |
| 490 | UNC13C |
| 491 | UNC5D |
| 492 | UQCC |
| 493 | USH2A |
| 494 | USP5 |
| 495 | USP8 |
| 496 | VAV1 |
| 497 | VCAN |
| 498 | VPS37A |
| 499 | VWA3B |
| 500 | WDR26 |
| 501 | WDR34 |
| 502 | WDR47 |
| 503 | WDR90 |
| 504 | WNK3 |
| 505 | XIRP2 |
| 506 | YLPM1 |
| 507 | ZC3H3 |
| 508 | ZC3HAV1L |
| 509 | ZCCHC11 |
| 510 | ZFYVE26 |
| 511 | ZIC3 |
| 512 | ZNF160 |
| 513 | ZNF18 |
| 514 | ZNF236 |
| 515 | ZNF334 |
| 516 | ZNF33A |
| 517 | ZNF418 |
| 518 | ZNF443 |
| 519 | ZNF444 |
| 520 | ZNF679 |
| 521 | ZNF695 |
| 522 | ZNF699 |
| 523 | ZNF709 |
| 524 | ZNF710 |
| 525 | ZNF91 |
